# Decreased admissions and change in arrival mode in patients with cerebrovascular events during the first surge of the COVID-19 pandemic

**DOI:** 10.1186/s42466-020-00094-w

**Published:** 2020-11-16

**Authors:** Carolin Hoyer, Lenja Weber, Vesile Sandikci, Anne Ebert, Michael Platten, Kristina Szabo

**Affiliations:** Department of Neurology and Mannheim Center for Translational Neuroscience, Heidelberg University, Medical Faculty Mannheim, Theodor-Kutzer-Ufer 1-3, 68135 Mannheim, Germany

**Keywords:** COVID-19, Emergency department, Stroke

## Abstract

**Background and purpose:**

Investigating clinical characteristics of patients presenting with cerebrovascular events during the pandemic may provide valuable insight into further understanding the phenomenon of decreased stroke admissions during the COVID-19 pandemic.

**Method:**

Data of patients presenting with a cerebrovascular event to the emergency department during weeks 12–17/2020 were compared to data from the respective weeks in 2019.

**Results:**

A significant reduction in the number of admissions by 35.9% (*p* = 0.005) was observed during the COVID-19 epoch. In addition, significantly more patients arrived by ambulance during the COVID-19 epoch (2019: 75.7%, 2020: 94.2%; *p* = 0.001).

**Conclusion:**

Our data may have implications as to how campaigns raising awareness for serious medical conditions in the context of the pandemic should be framed.

**To the Editor:**

Declining rates of admissions for cerebrovascular events (CVEs) and an impact on reperfusion therapy rates were observed during the first surge of the coronavirus disease 2019 (COVID-19) pandemic earlier this year. This applied regardless of the extent of COVID-19-related re-allocation of resources in different countries [1–5]. Avoidance behavior caused by fear of in-hospital infection was suggested as underlying this phenomenon [6], which in the case of undiagnosed or untreated strokes may have harmful consequences. Given the current increase in the number of COVID-19 cases and an incipient second wave, it is paramount to take appropriate measures to prevent this particular aspect of recent history from repeating. Obtaining detailed demographic and clinical information of patients presenting with CVEs during the pandemic may provide valuable information to this end.

We analyzed data of patients admitted for CVEs (transient ischemic attack (TIA), ischemic stroke, intracerebral hemorrhage) to the Department of Neurology, University Medical Centre Mannheim, Germany, in weeks 1–17/2020. Week 12/2020, when extended measures for social distancing were implemented, was designated as the beginning of the COVID-19 epoch. Poisson regression was used to test if the rate of admissions and reperfusion therapies for ischemic stroke changed as a function of year, epoch and year-by-epoch interaction (reflecting the impact of the pandemic). We found a significant reduction of the number of admissions due to a CVE during the COVID-19 epoch by 35.9% (rate ratio 0.64, 95% confidence interval (CI) 0.43–0.96, *p* = 0.005); Fig. 1). During the observational period of 2019 and 2020, 115 and 69 CVE patients, respectively, presented. The number of reperfusion therapies decreased non-significantly by 27.8% (rate ratio 0.72, 95% CI 0.44–1.19, *p* = 0.20): 23 intravenous thrombolyses (IVT) were performed in 2019, 11 in the respective period in 2020. Mechanical thrombectomy (with/without IVT), was performed in 9 and 8 patients, respectively. Group comparisons between weeks 12–17/2019 vs. 2020 were performed (Table 1): No differences were found regarding age, gender and vascular risk factors, NIHSS scores, rate of minor strokes, proportion of patients with TIA and symptom-to-door times. We also identified a change in admission mode between 2019 and 2020 with significantly more admissions via ambulance in 2020 (Fig. 1, *p* = 0.001).

**Fig. 1:**
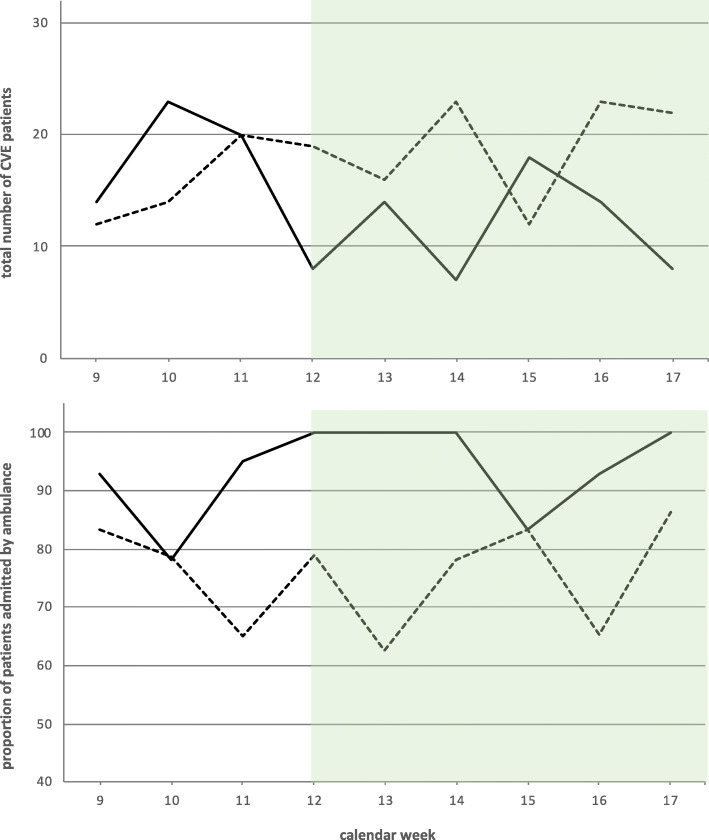
Admissions due to a CVE and arrival via ambulance during weeks 9-17 in years 2019 and 2020. CVE admissions decreased by 35.9% (upper panel) and admissions via ambulance increased by 18.5% (lower panel) during the COVID epoch in 2020. Pale green: COVID epoch. Dotted line: year 2019, solid line: year 2020. CVE = cerebrovascular event

**Table 1:** Demographics, vascular risk factors and index stroke data of patients admitted in calendar weeks 12–17 in 2019 and 2020

	2019 (***n*** = 115)	2020 (***n*** = 69)	***P*** value
***Demographics***			
Age, mean (±SD)	75.0 (12.3)	75.9 (12.5)	0.63
Gender, male, n (%)	51 (44.3)	35 (50.7)	0.40
***Vascular risk factors***			
Hypertension	85 (73.9)	56 (82.4)	0.19
Diabetes	34 (29.6)	17 (25.0)	0.51
Coronary heart disease	19 (16.5)	12 (17.6)	0.85
Peripheral vascular disease	6 (5.2)	1 (1.5)	0.26
Hyperlipoproteinemia	13 (11.3)	6 (8.8)	0.60
Smoking	21 (18.3)	10 (14.7)	0.54
***Index stroke***			
Mode of admission			**0.001**
Self-presenting, n (%)	28 (24.3)	4 (5.8)	
Admitted by ambulance, n (%)	87 (75.7)	65 (94.2)	
Symptom-to-door time, min (±SD)	235.7 (364.8)	244.9 (215.6)	0.88
Type of stroke			
Ischemic stroke, n (%)	112 (97.4)	67 (97.1)	1.00
TIA	31 (27.0)	12 (17.4)	0.14
Minor ischemic stroke^a, b^	32 (28.3)	23 (34.3)	0.40
Intracerebral hemorrhage, n (%)	3 (2.6)	2 (2.9)	1.00
NIHSS score, mean (±SD)	6.2 (7.8)	4.7 (5.6)	0.92
Reperfusion therapy, n (%)	32 (27.8)	19 (27.5)	0.97
IVT only	23 (20.0)	11 (15.9)	0.49
IVT door-to-needle time, min (±SD)	30.4 (13.5)	28.4 (6.4)	0.56
Mechanical thrombectomy (with or without IVT)	9 (7.8)	8 (11.6)	0.93

^a^minor stroke defined as NIHSS score between 1 and 3

^b^NIHSS score data available of 113 patients from 2019 and of 67 patients from 2020, percentages adapted accordingly

*NIHSS* National Institute of Health Scale, *IVT* Intravenous thrombolysis, *SD* Standard deviation, *TIA* Transient ischemic attack

Previous investigations found a decline of presentations in particular due to TIA or minor stroke during the pandemic [1, 4]. Moreover, changes in recanalization therapy rates were attributed to underlying presentation delays [5], which may in part be caused by patients’ indecisiveness as to whether they should present to a hospital. In a study of emergency department (ED) patients with neurological complaints, we previously identified a shift towards older and more severely affected patients with consecutive higher numbers of admissions to escalated care during the pandemic [7]. While we did not observe such disproportionate changes in demographics or stroke characteristics, we found a significant difference in admission modes: while overall fewer patients presented to the hospital during the COVID epoch in 2020, the vast majority of those who did arrived by ambulance. A UK investigation reported no significant reduction in ambulance call-outs for stroke during the lockdown, the authors concluded that patients do not hesitate to call an ambulance when experiencing stroke symptoms [8]. However, previous studies showed that a relevant portion of stroke patients arrive via other modes of transportation such as private or public transport [9], and that these patients are usually younger and less severely affected than patients arriving by ambulance [10]. Hence it may be precisely this subgroup of self-presenting or privately transported patients we have seen fewer of during the pandemic. We can only speculate about the reasons behind this observation but the perceived threat of contracting an infection while waiting in often crowded EDs may be a contributing factor. It bears mentioning that ours is a single-center study examining not the entire duration of the lockdown and beyond, thus limiting the generalization of our results. Longer-term analyses are needed to further elucidate on health-related behaviors pertaining to if, when and how emergency presentations are initiated in the context of the pandemic. In light of our findings, it appears nonetheless adequate to advise patients to call an ambulance when noticing acute-onset neurological deficits of any kind and to frame respective campaigns accordingly. Finally, it remains essential for healthcare providers to clearly communicate that stroke care remains a priority and that swift assessment in a safe environment is guaranteed regardless of mode of presentation.

## Data Availability

The datasets used and/or analyzed during the current study are available from the corresponding author on reasonable request.

## Abbreviations

COVID-19: Coronavirus disease 2019
CVE: cerebrovascular event
ED: emergency department
TIA: transient ischemic attack
